# Effect of age, sex, and body size on the blood biochemistry and physiological constants of dogs from 4 wk. to > 52 wk. of age

**DOI:** 10.1186/s12917-021-02976-w

**Published:** 2021-08-06

**Authors:** Ana Luisa Montoya Navarrete, Teódulo Quezada Tristán, Samuel Lozano Santillán, Raúl Ortiz Martínez, Arturo Gerardo Valdivia Flores, Lizbeth Martínez Martínez, María Carolina De Luna López

**Affiliations:** Department of Veterinary Clinic, Autonomous University of Aguascalientes, La Posta Zootécnica, Jesús María, C.P.20908 Aguascalientes, Mexico

**Keywords:** Blood chemistry, Puppies, Clinical pathology, Reference intervals, Physiological constants

## Abstract

**Background:**

Blood biochemistry and reference intervals help to differentiate between healthy and ill dogs as well as to provide information for the prognosis, evaluation, and monitoring; however, these intervals are often obtained from adult animals. It is essential to understand that puppies and adults are physiologically different, which justifies the need to obtain age-specific biochemical reference intervals. The aim of this research was to assess the potential effect of age, sex, body size, and their interaction on routine biochemical analytes and physiological constants (body temperature, heart rate, and respiratory rate). To carry out the research, we selected 197 healthy dogs of both sexes and different body sizes (small, medium and large) classified by age: group I (4–8 wk), group II (9–24 wk), group III (25–52 wk), and group IV (> 52 wk). The biochemical analysis included the measurement of the enzymatic activity of aspartate aminotransferase (AST), alanine aminotransferase (ALT), lactate dehydrogenase (LDH), gamma-glutamyl transferase (GGT), alkaline phosphatase (ALP), and the concentrations of cholesterol, triglycerides, total proteins, albumin, globulins, glucose, urea, and creatinine. Statistical analyses used analysis of variance (ANOVA) and a general linear model (GLM), which allows the comparison of multiple factors at two or more levels (*p* < 0.05).

**Results:**

The results of this study showed that ALT, total protein, albumin, globulin, urea, creatinine, and body temperature levels were lower in puppies than in adult dogs of group IV (*p* < 0.05), while the enzymatic activity of ALP, LDH, glucose concentration, and heart rate were higher. Whereas sex, body size and the interaction did not show a significant effect (*p* > 0.05).

**Conclusions:**

Some biochemical components are influenced by age. For this reason, this manuscript contributes with additional data for the clinical interpretation of blood biochemical results in puppies.

**Supplementary Information:**

The online version contains supplementary material available at 10.1186/s12917-021-02976-w.

## Background

Blood biochemistry is a useful diagnostic tool not only when overt pathologic conditions are investigated, but also when subtle abnormalities in organ systems are present [1]. Since the reference intervals (RIs) comprise values referred to the 95% of a healthy population, they have become one the most commonly used tools in clinical decisions [2]. However, only a few laboratories perform their reference studies, and most RIs consider values obtained from adult dogs; hence, those references should not be used to interpret test results in puppies [1, 3].

On the other hand, clinical signs of disease in puppies are often nonspecific. Often, the fact of not having specific reference ranges related to puppies could hinder the diagnosis [4–6]. Evaluating biochemical components is a valuable tool in these situations, but it could be hampered by the lack of proper RIs [5]. The physiologic differences between puppies and adult dogs require an age-specific biochemical RI, as in most species [5, 7]; thus, the RI for adults should not be used in puppies since it may lead to inaccurate interpretation of the results [2, 5, 8]. When interpreting clinical data obtained from dogs during their first year of life, growth-related alterations and metabolic changes in biochemical limits should be considered [6, 9].

Limited studies have examined the effect of age, sex, and body size on biochemistry variables in puppies, and most of these studies were performed with specific breeds in a controlled laboratory environment, where dogs experienced identical living and nutritional conditions [4, 8–10]. In most cases, the puppies’ history and physical examination are the same as for adults; however, there may be some variations in the physiological constants [11, 12].

For these reasons, with the aim to acquire new and more precise information to biuld puppies-specific RIs, this study assessed the potential effect of age, sex, and body size on routine serum biochemical analytes measured in four groups of dogs of different ages (4–8, 9–24, 25–52, and > 52 wk. of age).

## Results

A total of 197 blood samples were collected from dogs of different ages (35 from 4-8 wk, 51 from 9-24 wk, 23 from 25-52 wk, and 88 >52 wk of age), mixed and pure breed (106 female and 91 male dogs). Sixty-six dogs were mixed breeds, and 131 dogs were purebred. The main pure breeds identified were 20 French Poodle, 12 Schnauzer, 10 Pitbull, 10 Labrador, 10 Rottweiler, 9 Beagle, 8 German Shepherd, 8 Pug, 7 Chihuahua, 7 French Bulldog, 7 Boxer, 7 Yorkshire, 6 Cocker Spaniel, 5 Doberman, and 5 Siberian Husky. All dogs were grouped according to their body size: 90 small dogs, 50 medium dogs, and 57 large dogs. A variety of small, medium, and large dogs, as well as pure and mixed breeds, were represented in each age group. Fifteen blood samples were excluded from further biochemical analysis due to the presence of lipemia (9 samples), haemolysis (4 samples), or jaundice (2 samples). In addition, the values of seven dogs were excluded from the statistical analysis because of underlying subclinical diseases. Table 1 shows 90 T1 the mean, standard deviation, median, interquartile 91 range, and significant difference with a 95% confidence 92 level. It also shows the lower and upper limits of the reference intervals and the limits of reference calculated 94 with a 90% confidence interval. Data from all the dogs 95 were divided into four age groups: 4–8, 9–24, 25–52, 96 and > 52 wk. of age.

**Table 1:** Reference intervals by age

Variables	Distribution	(I) 4–8 wk. (*n* = 35)		(II) 9–24 wk. (*n =* 48)		(III) 25–52 wk. (*n* = 21)		(IV) > 52 wk. (*n* = 71)	
		Mean (SD) Median (IQR)	LL (90% CI) UL (90% CI)	Mean (SD) Median (IQR)	LL (90% CI) UL (90% CI)	Mean (SD) Median (IQR)	LL (90% CI) UL (90% CI)	Mean (SD) Median (IQR)	LL (90% CI) UL (90% CI)
AST (U/L)	G	39 (9.9) ^a^	18 (13–23)	40 (8.6) ^a^	21 (21–27)	38 (10.2) ^a^	16 (11–23)	38 (9.1) ^a^	21 (20–24)
		40 (16)	60 (55–64)	40 (11)	57 (54–57)	38 (15.5)	60 (53–66)	37 (16)	54 (52–54)
ALT (U/L)	NG	26 (8.8) ^a^	8 (4–12)	32 (9.1) ^b^	15 (14–20)	37 (7.6) ^b, c^	25 (24–27)	44 (12.6) ^c^	22 (21–26)
		26 (11)	45 (40–49)	31 (15.7)	54 (46–55)	36 (14.5)	60 (52–80)	41 (19)	70 (65–70)
LDH (U/L)	NG	244 (164.6) ^a^	43 (43–48)	73 (32.4) ^b^	25 (24–34)	63 (32) ^b^	6 (6–13)	67 (42) ^b^	9 (8–17)
		166 (228)	695 (526–861)	74 (55.5)	139 (131–140)	62 (51.5)	131 (112–150)	56 (39)	185 (143–187)
ALP (U/L)	NG	215 (71.6) ^a^	101 (91–115)	193 (39.2) ^a^	111 (110–123)	85 (36.7) ^b^	18 (10–36)	52 (23.3) ^c^	15 (15–23)
		186 (96)	394 (332–456)	187.5 (58)	281 (253–286)	90 (72)	179 (150–202)	47 (36)	112 (92–113)
GGT (U/L)	NG	5 (1.7) ^a^	2 (2–3)	5 (1.3) ^a^	2 (2–3)	5 (1.9) ^a^	2 (0.3–3)	6 (2.1) ^a^	2 (2–3)
		5 (2)	10 (9–12)	5 (2)	8 (7–8)	6 (2)	9 (8–10)	6 (4)	10 (9–10)
Total protein (g/dL)	NG	4.6 (0.6) ^a^	3.5 (3.3–3.7)	5.1 (0.7) ^b^	4.0 (4.0–4.0)	5.7 (0.8) ^b^	4.3 (4.1–4.6)	6.2 (0.9) ^c^	4.4 (4.2–4.8)
		4.4 (1.2)	6.4 (5.8–6.9)	5.1 (1.1)	7.1 (6.3–7.3)	5.5 (1.4)	7.9 (6.9–8.8)	6.3 (1.3)	8.2 (7.7–8.2)
Albumin (g/dL)	NG	2.5 (0.3) ^a^	1.9 (1.9–2.0)	2.7 (0.3) ^b^	2.1 (2.1–2.2)	3.1 (0.3) ^b, c^	2.2 (2.0–2.5)	3.1 (0.4) ^c^	2.4 (2.3–2.5)
		2.4 (0.4)	3.5 (3.2–3.9)	2.7 (0.6)	3.7 (3.4–3.8)	3.1 (0.5)	3.9 (3.7–4.2)	3.1 (0.6)	4.2 (3.9–4.4)
Globulins (g/dL)	NG	2.1 (0.6) ^a^	1.0 (0.9–1.2)	2.3 (0.6) ^a^	1.0 (1.0–1.4)	2.6 (0.6) ^a, b^	1.3 (0.9–1.8)	3.0 (0.7) ^b^	1.7 (1.6–1.9)
		2.1 (0.7)	3.5 (3.1–4.0)	2.3 (0.8)	3.9 (3.6–3.9)	2.7 (1.1)	4.0 (3.6–4.4)	3.0 (1.3)	4.3 (4.1–4.6)
Cholesterol (mg/dL)	G	169 (86.3) ^a^	62 (60–68)	199 (60.9) ^a^	92 (88–113)	185 (41.1) ^a^	97 (73–123)	186 (51) ^a^	80 (76–90)
		124 (144)	418 (309–493)	199 (105)	317 (306–318)	179 (54)	272 (245–299)	185 (55)	288 (283–291)
Triglycerides (mg/dL)	G	37 (18) ^a^	14 (13–16)	41 (13) ^a^	20 (20–23)	43 (14) ^a^	(12 (4–21)	45 (14) ^a^	14 (14–25)
		28 (22)	91 (65–114)	40 (18)	76 (67–76)	43 (16)	73 (64–82)	44 (18)	79 (66–80)
Glucose (mg/dL)	G	89 (21) ^a^	47 (38–56)	90 (18) ^a^	49 (47–61)	81 (21) ^a, b^	43 (37–53)	77 (21) ^b^	36 (31–50)
		89 (26)	132 (122–142)	92 (23)	135 (121–137)	78 (33)	131 (114–148)	74 (32)	122 (114–128)
Urea (mg/dL)	NG	23 (7) ^a^	12 (11–13)	27 (8.7) ^b^	9 (8–14)	36 (9.5) ^b, c^	15 (10–21)	34 (10) ^c^	14 (11–18)
		20 (12)	42 (35–48)	27 (10)	47 (43–48)	35 (15)	56 (50–62)	34 (15)	55 (49–56)
Creatinine (mg/dL)	NG	0.45 (0.09) ^a^	0.3 (0.3–0.3)	0.59 (0.16) ^b^	0.4 (0.4–0.4)	1.00 (0.30) ^c^	0.3 (0.0–0.5)	1.03 (0.25) ^c^	0.6 (0.5–0.6)
		0.45 (0.15)	0.7 (0.6–0.8)	0.54 (0.26)	1.0 (0.9–1.1)	0.99 (0.50)	1.6 (1.4–1.7)	1.05 (0.43)	1.6 (1.4–1.7)
Temperature (°C)	NG	37.9 (0.5) ^a^	36.7 (36.3–37)	38.5 (0.6) ^b^	37.3 (37.3–37.6)	38.7 (0.4) ^b, c^	37.7 (37.5–38)	38.8 (0.4) ^c^	37.9 (37.9–38.1)
		38.0 (0.7)	39 (38.8–39.2)	38.5 (0.8)	39.6 (39.4–39.6)	38.7 (0.7)	39.6 (39.3–39.7)	38.9 (0.7)	39.7 (39.5–39.7)
Heart rate (bpm)	NG	155 (28.4)^a^	86 (64–107)	145 (33)^a^	85 (84–92)	129 (26)^a, b^	74 (58–90)	125 (27)^b^	80 (80–89)
		160 (44)	207 (196–216)	140 (38.5)	200 (200–200)	132 (28)	185 (168–202)	120 (40)	180 (180–180)
Breathing frequency (bpm)	NG	30 (6.6)^a^	15 (13–19)	33 (5.9)^a^	22 (22–24)	34 (6.6)^a^	20 (16–24)	32 (7)^a^	15 (15–20)
		30 (12)	44 (41–46)	31 (8)	44 (40–44)	36 (12)	44 (44–44)	32 (16)	44 (44–46)

*CI* confidence interval; °*C* degrees Celsius *G* gaussian; *IQR* inter quartile range; *LL* low limit; *NG* not gaussian; *SD* standard deviation; *UL* upper limit. Groups that do not share a letter are significantly different (a, b, c) *p* < 0.05

The effect of age, sex, and body size on biochemical serum variables (AST, ALT, LDH, ALP, GGT, total protein, albumin, globulins, cholesterol, triglycerides, glucose, urea, and creatinine) was assessed. Additionally, the effects on body temperature (rectal), heart rate, and respiratory rate were evaluated.

The following statistical data are reported in the results: between-groups degrees of freedom, the within-groups degrees of freedom (separated by a comma). After that the *F* statistic (rounded off to two decimal places) and the significance level (*p*). Mean and standard deviation are most clearly presented in parentheses.

### Effect of age

**Fig. 1:**
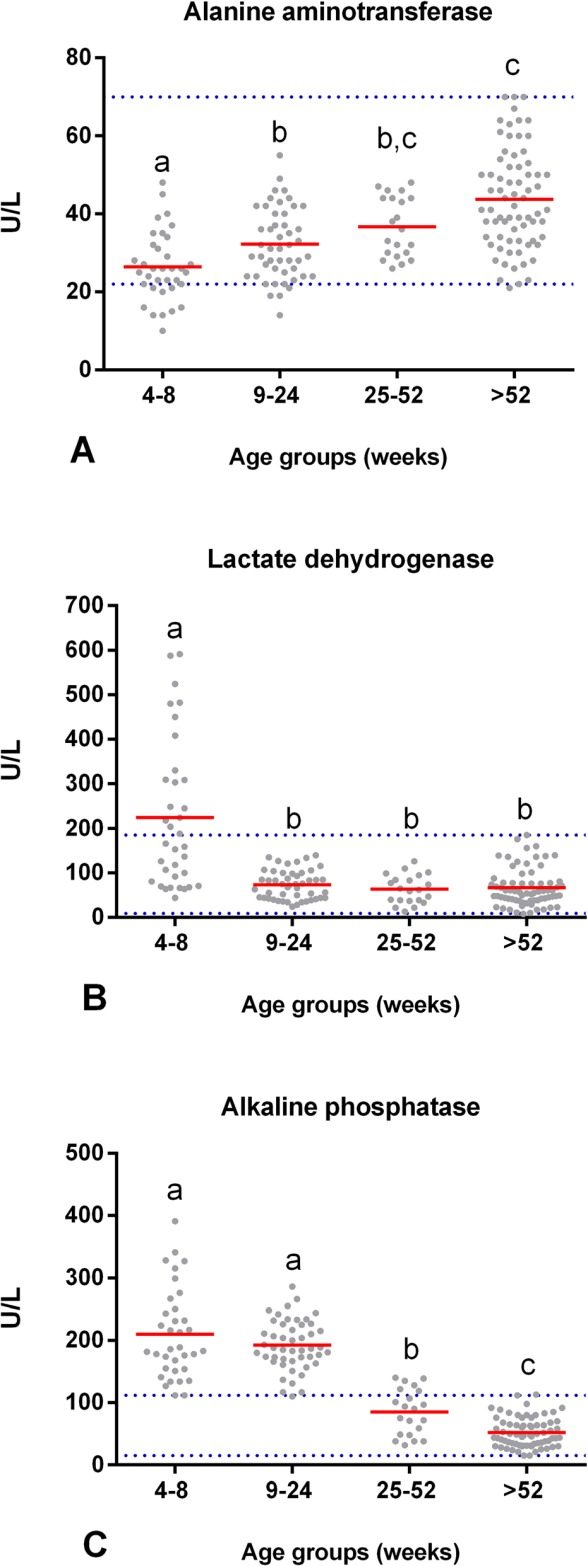
Enzymatic blood activity at different stages of the dog’s life. Comparison of the means (red line) of (**A**) ALT, (**B**) LDH, and (**C**) ALP between groups of clinically healthy dogs of different ages: group I (*n* = 35, 4–8 wk), group II (*n* = 48, 9–24 wk), group III (*n* = 21, 25–52 wk), and group IV (*n* = 71, > 52 wk). Horizontal blue dotted lines represent the RIs for adult dogs with a 90% CI (see Table 1). Means that do not share a letter are significantly different (*p* < 0.05)

No significant differences were observed in the AST activity (*F* (3,158) = 0.59, *p* = 0.62). In contrast, the enzymatic activity of ALT had an effect on age (*F* (3,158) = 22.11, *p* = 0.00). The average ALT activity in puppies from 4 to 8 wk. of age was 26 U/L (*SD* = 8.8), which was significantly lower than the enzymatic activity in dogs from 9 to 24 wk. of age (*p* = 0.01), 25–52 wk. of age, and > 52 wk. of age (*p* = 0.00). Moreover, the ALT activity was significantly lower in dogs from 9 to 24 wk. of age (*M* = 32 U/L, *SD* = 9.1) than in the adult group IV (*p* = 0.00). Furthermore, the ALT activity in young dogs of 25–52 wk. of age (*M* = 37 U/L, *SD* = 7.6) was not significantly different compared to the enzymatic activity in adult dogs > 52 wk. of age (*M* = 44 U/L, *SD* = 12.6; *p* = 0.17). Therefore, Fig. 1 shows that the ALT activity was lower in puppies of 4–24 wk. of age; approximately from 25 wk. of age, the enzymatic activity was similar to that in adults.

Age had a significant effect on the enzymatic activity of LDH (*F* (3,158) = 19.44, *p* = 0.00). The average LDH activity in puppies from 4 to 8 wk. of age was 244 U/L (*SD* = 164.6), which was significantly higher than the enzymatic activity in groups II (*M* = 73 U/L, *SD* = 32.4), III (*M* = 63 U/L, *SD* = 32), and IV (*M* = 67 U/L, *SD* = 42) (*p* = 0.00). Our research showed that LDH activity decreased as age increased, and the values stabilized approximately at 9 wk. of age (Fig. 1).

Age had a significant effect on the enzymatic activity of ALP (*F* (3,158) = 165.04, *p* = 0.00). The average ALP in puppies from 4 to 8 wk. of age was 215 U/L (*SD* = 71.6), which was significantly greater than the enzymatic activity of groups III and IV (*p* = 0.00). The ALP activity of group II (*M* = 193 U/L, *SD* = 39.2) also showed significantly higher activity than that of groups III (*p* = 0.00) and IV (*p* = 0.00). Finally, the ALP enzymatic activity of group III (*M* = 85 U/L, *SD* = 36.7) was significantly greater than that of group IV (*M* = 52 U/L, *SD* = 23.3; *p* = 0.00). These results suggest that ALP enzymatic activity decreases as the age of dogs increases. The serum ALP activity at 4–24 wk. of age in puppies was four times higher than that in adults; in young dogs from 25 to 52 wk. of age, the ALP activity was almost two times higher than the activity in adults (Fig. 1). On the other hand, age did not have a significant effect on the enzymatic activity of GGT (F (3,158) = 1.33, *p* = 0.26).

**Fig. 2:**
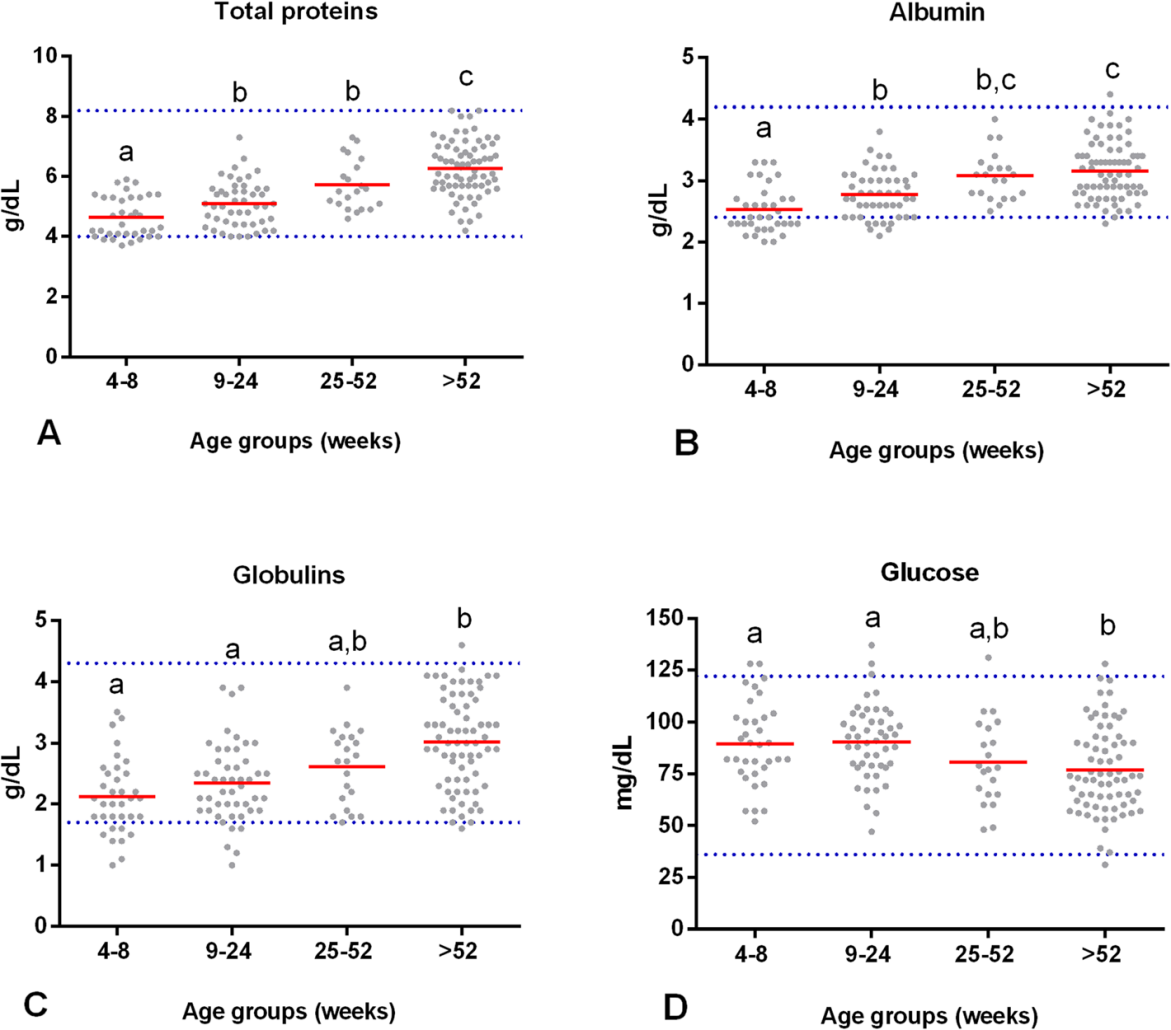
The serum concentration of some analytes at different stages of the dog’s life. Comparison of the mean (red line) (**A**) protein total, (**B**) albumin, (**C**) globulins, and (**D**) glucose levels between groups of clinically healthy dogs of different ages: group I (*n* = 35, 4–8 wk), group II (*n* = 48, 9–24 wk), group III (*n* = 21, 25–52 wk), and group IV (*n* = 71, > 52 wk). Horizontal blue dotted lines represent the RIs for adult dogs with a 90% CI (see Table 1). Means that do not share a letter are significantly different (*p* < 0.05)

Age had a significant effect on the concentration of total proteins (*F* (3,158) = 32.21, *p* = 0.00). The average serum total protein concentration in puppies from 4 to 8 wk. of age (*M* = 4.6 g/dL, *SD* = 0.6) was significantly lower than that in dogs from 9 to 24 wk. of age (*p* = 0.02), 25–52 wk. of age, and > 52 wk. of age (*p* = 0.00). Furthermore, that average was significantly lower in dogs from 9 to 24 wk. of age (*M* = 5.1 g/dL, *SD* = 0.7) than in adults of group IV (*p* = 0.00). Finally, the concentration of the group from 25 to 52 wk. of age (*M* = 5.7 g/dL, *SD* = 0.8) was lower than that in dogs of > 52 wk. of age (*M* = 6.2 g/dL, *SD* = 0.9; *p* = 0.03). Therefore, these results show in Fig. 2 that the serum total proteins in puppies from 4 to 8 wk. of age are lower than for dogs from 9 to 52 wk. of age, they begin to increase until they stabilize after 52 wk. of age.

A significant effect of age on the albumin concentration was observed (*F* (3,158) = 21.38 *p* = 0.00). The albumin concentration in group I (*M* = 2.5 g/dL, *SD* = 0.7) was significantly lower than the concentration in groups II (*M* = 2.7 g/dL, *SD* = 0.3; *p* = 0.01), III (*M* = 3.1 g/dL, *SD* = 0.3; *p* = 0.00), and IV (*M* = 3.1 g/dL, *SD* = 0.4; *p* = 0.00). In the group II puppies from 9 to 24 wk. of age, the concentration was significantly lower than that of group IV (*p* = 0.00). These results show that the serum albumin concentration increases as a dog’s age advances. The albumin concentration is lower in puppies from 4 to 8 wk. of age than in dogs at 9 wk. of age, it begins to increase; approximately at 25 wk. of age, the albumin concentration stabilizes at adult values (Fig. 2).

Age had a significant effect on the concentration of globulins (*F* (3,158) = 12.89, *p* = 0.00). The average serum globulins in puppies from 4 to 8 wk. of age were 2.1 g/dL (*SD* = 0.6) and 2.3 g/dL (*SD* = 0.6) in puppies from 9 to 24 wk. of age, which were significantly lower than those in dogs from > 52 wk. of age (*M* = 3 g/dL, *SD* = 0.7; *p* = 0.00). The average globulin concentration in young dogs from 25 to 52 wk. of age was 2.6 g/dL (*SD* = 0.6), which was the same as that in groups I (*p* = 0.25), II (*p* = 0.94), and IV (*p* = 0.06). Therefore, these results indicate that the serum globulin concentration in puppies from 4 to 24 wk. of age is lower than in dogs from 25 wk. of age, it begins to increase until it stabilizes after 52 wk. of age (Fig. 2).

No significant effect of age was observed on the concentration of cholesterol (*F* (3,158) = 1.49, *p* = 0.22) and triglycerides (*F* (3,158) = 2.52, *p* = 0.06). A significant effect of age on the glucose concentration was observed (*F* (3,158) = 4.14, *p* = 0.01). This concentration was significantly higher in puppies from 4 to 8 wk. (*M* = 89 mg/dL, *SD* = 21, *p* = 0.02) and from 9 to 24 wk. of age (*M* = 90 mg/dL, *SD* = 18, *p* = 0.00) than in adults > 52 wk. (*M* = 77 mg/dL, *SD* = 21). This result suggests that there is a decrease in glucose concentration as the age of the dog increases. In addition, it was established that puppies reached the glucose levels of an adult approximately at 25 wk. of age (*M* = 81 mg/dL, *SD* = 21) (Fig. 2).

**Fig. 3:**
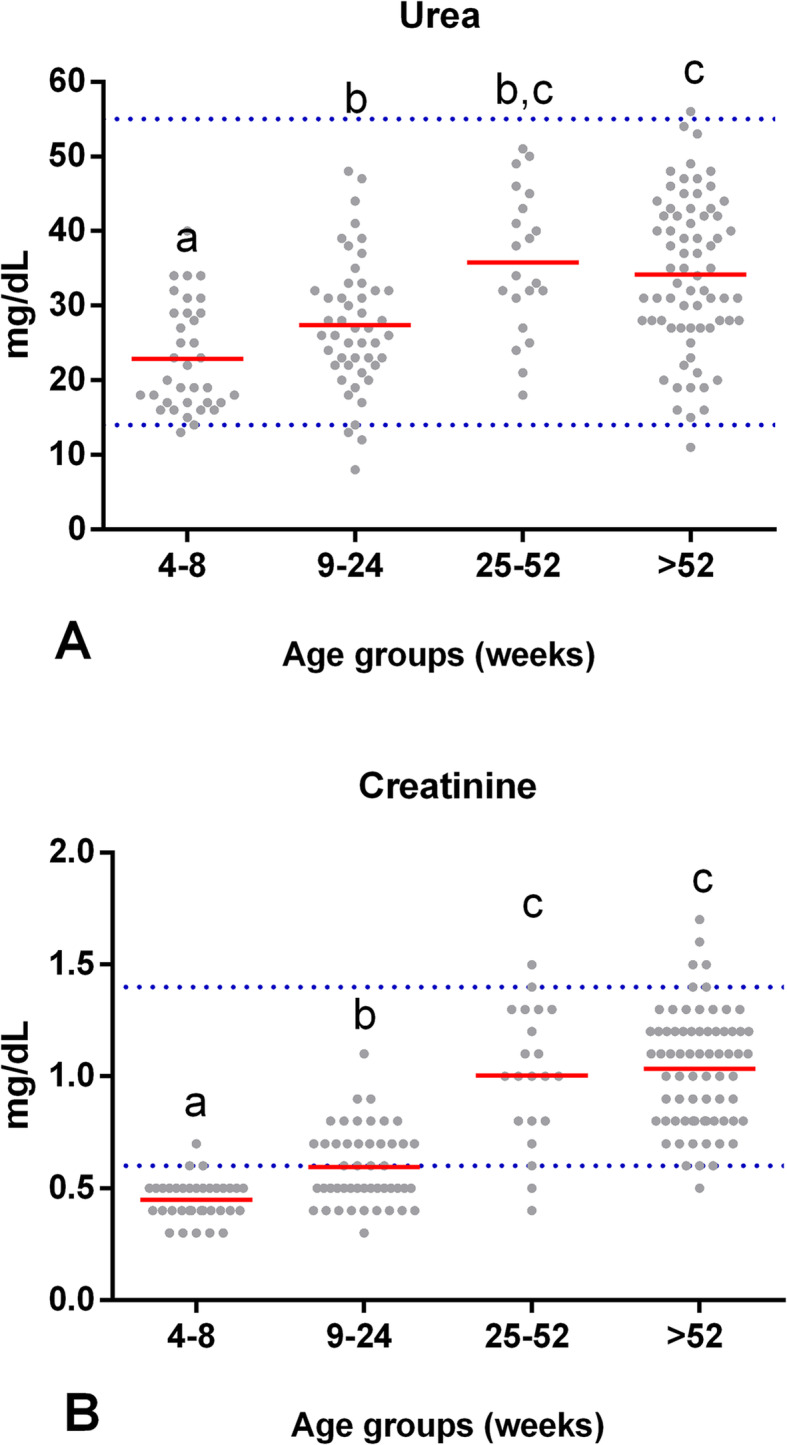
The serum concentration of some analytes at different stages of the dog’s life. Comparison of the mean (red line) (**A**) urea and (**B**) creatinine levels between groups of clinically healthy dogs of different ages: group I (*n* = 35, 4–8 wk), group II (*n* = 48, 9–24 wk), group III (*n* = 21, 25–52 wk), and group IV (*n* = 71, > 52 wk). Horizontal blue dotted lines represent the RIs for adult dogs with a 90% CI (see Table 1 for age-specific reference intervals). Means that do not share a letter are significantly different (*p* < 0.05)

Our research found an effect of age on serum urea concentration (*F* (3,158) = 14.84 *p* = 0.00). The urea concentration was significantly lower in puppies from 4 to 8 wk. of age (*M* = 23 mg/dL, *SD* = 7) than in puppies from groups II (*p* = .047), III (*p* = 0.00), and IV (*p* = 0.00). Similarly, puppies from 9 to 24 wk. of age (*M* = 27 mg/dL, *SD* = 8.7) showed significantly lower urea levels than dogs from group IV (*p* = 0.00). Furthermore, the urea concentration in young dogs from 25 to 52 wk. of age (*M* = 36 mg/dL, *SD* = 9.5) was equal to that of adult dogs > 52 wk. of age (*M* = 34 mg/dL, *SD* = 10; *p* = 0.99). Therefore, Fig. 3 shows that the urea concentration was lower in puppies from 4 to 24 wk. of age compared to adults, and it stabilized approximately in wk. 25.

Age had a significant effect on the concentration of creatinine (*F* (3,158) = 78.92 *p* = 0.00). In puppies from 4 to 8 wk. of age (*M* = 0.45 mg/dL, *SD* = 0.09), the creatinine concentration was lower than the concentrations in groups II, III, and IV (*p* = 0.00). Creatinine levels in puppies from 9 to 24 wk. of age (*M* = 0.59 mg/dL, *SD* = 0.16) were lower than those from groups III and IV (*p* = 0.00). Furthermore, creatinine in young dogs from 25 to 52 wk. of age (*M* = 1.00 mg/dL, *SD* = 0.30) was similar to the concentration in adult dogs > 52 wk. of age (*M* = 1.03 mg/dL, *SD* = 0.25; *p* = 0.81), while creatinine concentration in puppies from 4 to 24 wk. of age was lower. Approximately from 25 wk. of age, the concentration was the same as that of adults (Fig. 3).

**Fig. 4:**
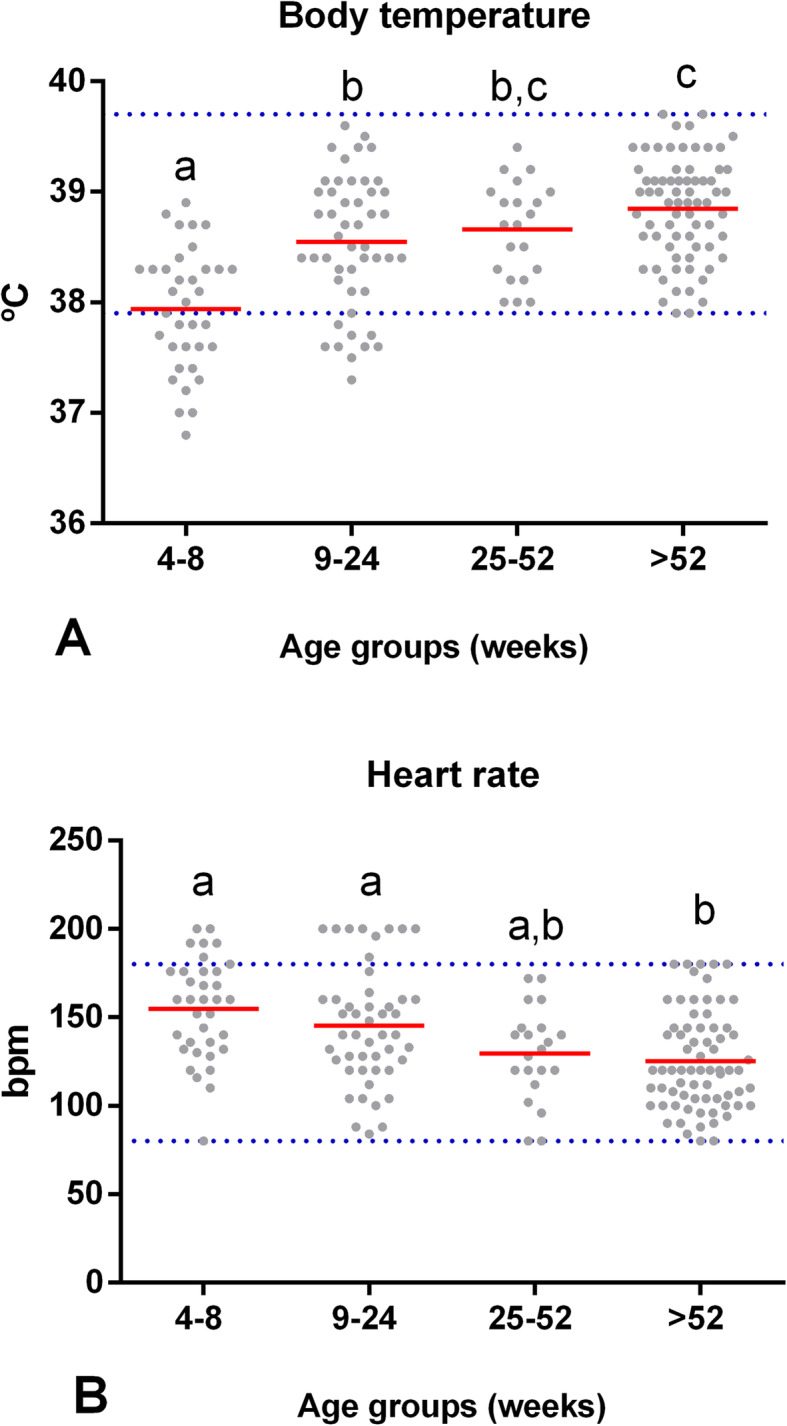
Values of the physiological constants at different stages of the dog’s life. Comparison of the mean (red line) (**A**) body temperature and (**B**) heart rate between groups of clinically healthy dogs of different ages: group I (*n* = 35, 4–8 wk), group II (*n* = 48, 9–24 wk), group III (*n* = 21, 25–52 wk), and group IV (*n* = 71, > 52 wk). Horizontal blue dotted lines represent the RIs for adult dogs with a 90% CI (see Table 1 for age-specific reference intervals). Means that do not share a letter are significantly different (*p* < 0.05)

A significant effect of age on body temperature was observed (*F* (3,158) = 18.62, *p* = 0.00). Body temperature in puppies from 4 to 8 wk. of age (*M* = 37.9 °C, *SD* = 0.5) was lower than the temperature obtained from groups II, III (*M* = 38.7 °C, *SD* = 0.4), and IV (*M* = 38.8 °C, *SD* = 0.4) (*p* = 0.00). Furthermore, in puppies from 9 to 24 wk. of age (*M* = 38.5 °C, *SD* = 0.6), their body temperature was lower than that of adults > 52 wk. of age (*p* = 0.01). These results indicate that dogs from 4 to 24 wk. of age have a lower body temperature. Figure 4 shows that approximately in dogs from 25 wk. of age, the temperature began to increase and was similar to that in adults > 52 wk. of age (*p* = 0.53).

Age had a significant effect on heart rate (*F* (3,158) = 5.44, *p* = 0.00). In puppies from 4 to 8 (*M* = 155 bpm, *SD* = 28.4; *p* = 0.00) and from 9 to 24 (*M* = 145 bpm, *SD* = 33; *p* = 0.01) wk. of age, the heart rate was higher than that of adults > 52 wk. (*M* = 74 bpm, *SD* = 33). Additionally, in the group from 25 wk. of age, the heart rate was similar to that of adults (*p* = 0.86). Therefore, our study shows a decrease in the heart rate as the animal’s age increases (Fig. 4). Finally, there was no statistically significant effect of age on the respiratory rate (*F* (3,158) = 1.21, *p* = 0.31).

### Effect of sex

No significant effect of sex was observed on the enzymatic activity of AST (*F* (1,158) = 2.98, *p* = 0.09), ALT (*F* (1,158) = 1.24, *p* = 0.28), LDH (*F* (1,158) = 1.74, *p* = 0.19), ALP (*F* (1,158) = 2.75, *p* = 0.10), or GGT (*F* (1,158) = 0.12, *p* = 0.73). There was no significant effect of sex on the serum concentrations of total proteins (*F* (1,158) = 0.14, *p* = 0.70), albumin (*F* (1,158) = 0.45, *p* = 0.50), globulins (*F* (1,158) = 0.62, *p* = 0.43), cholesterol (*F* (1,158) = 0.44, *p* = 0.51), triglycerides (*F* (1,158) = 1.11, *p* = 0.29), glucose (*F* (1,158) = 0.02, *p* = 0.90), urea (*F* (1,158) = 0.50, *p* = 0.48), or creatinine (*F* (1,158) = 1.40, *p* = 0.24). Sex did not have a significant effect on variable physiological constants: body temperature (*F* (1,158) = 0.71, *p* = 0.40), heart rate (*F* (1,158) = 2.86, *p* = 0.09), and respiratory rate (*F* (1,158) = .80, *p* = 0.37).

### Effect of body size (small, medium and large)

No significant differences were observed in the enzymatic activity of AST (*F* (3,158) = 1.34 *p* = 0.26), ALT (*F* (3,158) = 0.87, *p* = 0.46), LDH (*F* (3,158) = 2.61, *p* = 0.05), ALP (*F* (3,158) = 1.32, *p* = 0.27), or GGT (*F* (3,158) = 0.77, *p* = 0.51). There was also no significant effect of body size on the serum concentrations of total proteins (*F* (3,158) = 0.21, *p* = 0.88), albumin (*F* (3,158) = 0.50, *p* = 0.68), globulins (*F* (3,158) = 0.59, *p* = 0.63), cholesterol (*F* (3,158) = 2.39, *p* = 0.07), triglycerides (*F* (3,158) = 1.85, *p* = 0.14), glucose (*F* (3,158) = 1.36, *p* = 0.26), urea (*F* (3,158) = 1.36 *p* = 0.26) or creatinine (*F* (3,158) = 0.76 *p* = 0.54). Body size had no significant effect on physiological constants: body temperature (*F* (3,158) = 0.47, *p* = 0.70), heart rate (*F* (3,158) = 1.94, *p* = 0.13), or respiratory rate (*F* (3,158) = 1.77, *p* = 0.15).

### Age, sex and body size interaction

No statistically significant effects were observed in the enzymatic activity of AST (*F* (9,158) = 0.76, *p* = 0.65, ALT (*F* (9,158) = 0.87, *p* = 0.55), LDH (*F* (9,158) = 1.41, *p* = 0.19), ALP (*F* (9,158) = 0.44, *p* = 0.91), or GGT (*F* (9,158) = 1.54, *p* = 0.14). There was no significant effect of the interaction on the serum concentration of total proteins (*F* (9,158) = 0.81, *p* = 0.61), albumin (*F* (9,158) = 0.78, *p* = 0.64), globulins (*F* (9,158) = 0.77, *p* = 0.64), cholesterol (*F* (9,158) = 0.79, *p* = 0.62), triglycerides (*F* (9,158) = 1.29, *p* = 0.25), glucose (*F* (9,158) = 0.54, *p* = 0.84), urea (*F* (9,158) = 1.92, *p* = 0.05) or creatinine (*F* (9,158) = 0.79, *p* = 0.63). Body temperature (*F* (9,158) = 0.69, *p* = 0.71), heart rate (*F* (9,158) = 0.55, *p* = 0.84), and respiratory rate (*F* (9,158) = 0.74, *p* = 0.67) also did not show significant effects.

## Discussion

Regarding the effect of age on the enzymatic activity of AST and ALT, research shows contrasting results. Within this study population, no significant effect was observed for AST activity, similar to previous studies in puppies from 16 to 60 days of age [5] and in dogs from > 10 months to < 8 years of age [13]. Nonetheless, other research did find age-related differences in AST enzyme activity in dogs from 8 to 52 wk. of age [9] and in puppies from 0 to 3 and 6 months of age [10]. This study shows that the enzymatic activity of ALT tends to increase with age. The effect of age on ALT activity is consistent with prior results [9, 10, 14, 15]. However, some studies did not report significant differences in their results in puppies when compared to adults [4, 5]. These changes in ALT activity derive from physiological variations related to age [14]. Despite the early embryogenic differentiation of the liver, many of its metabolic functions are incompletely developed at birth. The foetal liver has a lower capacity for gluconeogenesis, glycogen storage, bile acid metabolism, detoxification, and elimination processes, making it more susceptible to toxins and transplacental and postnatal infections that may not have consequences in adults [16]. Although ALT predominates in the liver, AST is also present in cardiac muscle, skeletal muscle, liver, and kidneys. The age-dependent activities of both enzymes appear to correlate well with tissue growth [9].

The enzymatic activity of LDH evaluated in the present study was higher in puppies from 4 to 8 wk. old than in the other phases. However, a study conducted in 2013 did not observe age-related differences in LDH enzyme activity [10]. In puppies, LDH activity is at the highest levels during suckling, likely because of the enhanced use of lactose as a glucose precursor during the neonatal period. Adult values are obtained soon postweaning [1]. Early increases in LDH activity probably reflect muscle trauma associated with delivery [17]. Nevertheless, information about this enzyme is limited, and few studies have included it in their research.

ALP enzyme activity remained elevated from wk. 4 to wk. 52 compared to adults, but it began to decrease after wk. 25. Numerous reports indicate that in young animals, there is an increase in ALP activity compared to adults [5, 6, 10, 11]. This is the result of the activity of the ALP bone isoenzyme, which is increased in serum during bone development and growth [1, 5]. While no significant effect of age was observed on GGT activity, postweaning GGT values slightly decreased to below adult values; then, they increased to reach adult values at approximately 6 months of age. Moreover, in puppies postweaning, serum GGT activity is believed to reflect the enzyme derived from other tissues, mainly the liver [1]. On the other hand, due to the high levels of the activity of ALP and GGT contained in colostrum, the evaluation of these enzymes in serum or plasma provides some important information about the transfer status of passive immunity in puppies when used as a marker of the adequate ingestion of colostrum. However, these differences are short-lived and are determined within the first 2 wk. of age [1, 6].

Age showed a significant effect on the serum concentrations of total proteins and albumin. These results agree with previous research [4, 5, 9, 14]. Age-associated increases in the concentrations of total proteins and albumin are attributed to normal immune stimulation, which results in an elevated globulin fraction and albumin production derived from improved liver function and intestinal absorption [1]. The lower serum concentration of albumin in dogs younger than 24 wk. of age derives from the increased demand for albumin during this phase of intense growth [15]. In the same way, the concentration of globulins showed a tendency to increase as age advanced. This result agrees with previous studies [5, 15]. Puppies are born hypogammaglobulinaemic with only a small amount of IgG and IgM and no detectable IgA in serum at birth. Therefore, the total protein concentration in puppies is initially low, particularly precolostral intake [1]. The total protein concentration then steadily increases during the first year of life, and it stabilizes from 1 year old onward. On the other hand, during the first 6 wk. of life, a decrease in globulin concentration occurs due to the degradation of maternal antibodies; meanwhile, an increase in albumin concentration occurs because of normal liver function development [1].

No effect of age was observed on the concentration cholesterol and triglycerides. While a study from 2008 did not find an effect of age on triglyceride levels, the cholesterol concentration was higher in dogs from < 52 wk. of age [18]. Other studies from 2012 and 2015 found no significant difference in the cholesterol concentration of puppies and adults [4, 5]. However, in a longitudinal study conducted in 2016 in Labrador and Miniature Schnauzer dogs, blood samples were obtained from 8 to 52 wk. of age, and they found higher concentrations of cholesterol at wk. 26 and triglycerides at wk. 20 and 36 [9]. The neonatal liver has a lower capacity to synthesize triglycerides and cholesterol, so neonates depend on the lipids absorbed through the diet, which is why breastfeeding is an important source of lipids in new-borns [6].

The glucose concentration was higher in puppies from 4 to 24 wk. of age than in adults. Some research developed in dogs of different ages showed that the concentration of glucose decreased with growth [1, 5, 10]. A study found blood glucose values to be similar to those of adults on day 4 but significantly higher at all other time points, with a peak at wk. 4 [8]. However, in another study, there was no significant difference in the glucose concentration of puppies compared to adult dogs [4]. Glucose in the blood is closely regulated and normally maintained by three major mechanisms: intestinal absorption, hepatic production, and, to a lesser degree, renal production [1]. In young animals, there is a reduced potential for gluconeogenesis and glycogenolysis [1]. Furthermore, the inability of puppies to recover quickly from hypoglycaemia or hyperglycaemia can be attributed to their insensitivity to endogenous insulin and the low response of counterregulatory hormones (epinephrine and cortisol) [17]. Although glucose regulation improves with age, puppies up to 16 wk. of age should be considered predisposed to hypoglycaemia when they are anorexic or dehydrated [6].

Blood urea and creatinine concentration are the most commonly assessed indices of glomerular filtration in mammals. As these components are freely filtered by the glomerulus, any reduction in the glomerular filtration rate (GFR) results in increases in the concentration of these analytes in serum. However, both urea and creatinine serum concentrations could be influenced by other body systems, which may affect their rate of production and their rates of excretion. Age variations have been noted for both parameters [1]. The urea concentration evaluated in the present study increased with advancing age. These results agree with previous studies [4, 8, 9]. One previous study found a lower concentration of urea in puppies compared to adult values, although it did not find statistically significant differences [5]. This increase may be due to an increase in protein metabolism, because the puppies are in the growth stage [14]. Some proposals explain the low urea concentration in puppies as stemming from the increment in protein synthesis by the influence of growth hormone or the increase in metabolic status with the glomerular filtration rate (GFR) [1]. The GFR increases with age postnatally. Glomerular capillary surface area and pore density increase between the first and sixth wk. after birth. Studies suggest that GFR and renal blood flow increase up to 11 wk. of age in the puppy before reaching adult levels [19]. Serum creatinine levels also tended to increase with advancing age. Our results are consistent with some studies [4, 5, 10, 20]. Moreover, the lower creatinine in young animals, in relation to adults, correlates with the smaller body size and lower muscle mass [1]. It is important to have an age-specific reference interval to identify an increase in creatinine concentration in puppies.

Age had an effect on body temperature and heart rate. In the second and third wk. of life, before the puppies are in transition phase from crawling and walking properly, normal body temperature oscillates from 37.0° to 38.2 °C [12]. However, one previous study mentioned that weaned puppies and young dogs have the same normal body temperature as adult animals [11]. The mean heart rate in healthy puppies is approximately 220 beats per minute (bpm) during the first wk. of life [12]. On the other hand, the respiratory rate had no significant age-related effect. The respiratory rate is the same as that in adults by 4 wk. of age [11].

Regarding sex, no significant effect was observed. These results coincide with some studies from 2013 and 2016 where researchers did not find an effect of sex on the enzymatic activity of ALT, AST, LDH, ALP, and the concentration of total protein, albumin, cholesterol, triglycerides, glucose, creatinine, and urea [9, 10]. In other research from 2008, they did not find an effect of sex on the concentration of triglycerides, yet the cholesterol concentration was higher in females than in males [18]. Some researchers have reported statistically significant differences according to sex in some biochemical parameters, such as cholesterol and aminotransferases [13]. However, our research shows that for the tested variables in this study, there is no need to establish sex-specific RIs, but it is important to consider its effect when interpreting results.

Dog body size no significant effect was observed. These results coincide with a study of medium- and large-sized dogs found no significant effect of body size on biochemical variables [21]. However, one previous study of small dogs reported a breed effect evident in the concentration of urea, total proteins, albumin, glucose, and the enzymatic activity of ALT [13].

Regarding the physiological constants statistically evaluated in this study, we did not observe any effect of sex or body size. Nevertheless, it is necessary to have a greater number of studies that provide information about the effects related to age, sex, and body size on these variables.

Finally, age, sex, and body size did not have any significant interaction on the evaluated variables. However, one study reported a significant interaction of body size and age on biochemical tests of young Labrador Retrievers and Miniature Schnauzers between 8 and 52 wk. of age [9]. Another study from 2014 observed a significant interaction between body size and sex for plasma cholesterol concentration and ALT enzymatic activity [13].

Our study showed that enzymatic activity ALT, the concentration of total protein, albumin, globulin, urea, creatinine, and body temperature levels were lower in puppies at 4–8 wk. of age than in adult dogs. For most of these variables, by 25 wk. of age, their values were similar to those of adult dogs; these variables began to increase until reaching adult values except for total proteins, where their concentration began to increase from 9 wk. of age and remained stable after 52 wk. of age. In contrast, the enzymatic activity of ALP, LDH, glucose concentration, and heart rate were higher in puppies from 4 to 24 wk. of age than in adult dogs.

Moreover, the values of ALP activity, glucose concentration, and heart rate began to decrease approximately from wk. 25, while the enzymatic activity of LDH decreased from wk. 9. The results of enzymatic activity AST, GGT, the concentration of cholesterol, triglycerides, and the respiratory rate did not show an effect of age.

One limitation of this study is the sample size in each age group when determining reference intervals. The American Society of Veterinary Clinical Pathology recommends at least 120 samples. Some sample sizes were < 40 dogs (35 from 4 to 8 wk., 48 from 9 to 24 wk., 21 from 25 to 52 wk., and 71 > 52 wk. of age). However, reference intervals determined from smaller sample sizes are commonplace and often unavoidable in veterinary medicine [2]. According to Friedrichs (2012) when ≥20 and < 40 reference samples are available, RI should be calculated by methods that are robust (distribution-independent) or parametric (if normality can be established). To highlight the uncertainty inherent with small sample sizes, 90% CI should be calculated. In addition, should be reported mean or median, and minimum and maximum values to allow informed clinical decision-making (Table 1) [2, 22].

Notably, in this study, a direct sampling method was approached applying inclusion and exclusion criteria [2]. Preparation of reference individuals, sample collection, sample handling, and sample processing were performed in a standardized manner. Samples were analyzed using methods that are stringently monitored with appropriate quality control procedures [23, 24]. Criteria were established for rejection of samples due to their poor quality. Results were monitored in real-time so that errors for detecting and perform re-measurement [2].

## Conclusion

Thus, it is evident that some biochemical components are influenced by age. For this reason, our research offers additional data that can help veterinary clinicians in evaluating the biochemical results obtained from puppies. Therefore, we suggest more research to keep the information up to date, including the evaluation of other biochemical, vital, and haematological variables.

## Methods

### Study area and population

This study was carried out in compliance with the provisions established in the Ethics regulations for the use of animals in teaching and research at the Autonomous University of Aguascalientes, Code: DI-PL-NO-37 [25]. A non-experimental transverse design was used [26].

We selected 197 healthy dogs of different sexes and sizes classified by age: group I (4–8 wk), group II (9–24 wk), group III (25–52 wk), and group IV (> 52 wk). The breeds were grouped according to their body size: small-sized (< 9.5 kg), medium-sized (9.5–22.7 kg) and large-sized dogs (> 22.7–54.5 kg) [27].

Animals were assessed as healthy on the basis of a complete physical examination and history. The collection of the medical history and the physical examination were carried out by a veterinarian who specializes in clinical medicine of small species at the Veterinary Hospital of the Autonomous University of Aguascalientes and private clinics. A questionnaire (Additional file 1) related to health, living environment, activity, behavioural changes, nutrition, vaccination, parasite control, and medical history was administered [2]. The questionnaire was reviewed with the owner, and additional questions were asked if required. During this time, the dog was allowed to freely explore the examination room. An animal information form in Additional file 1 (documenting date of birth, sex, breed, weight, body size, diet, reproductive status, vital signs) was also obtained for each dog before inclusion in the study [11, 12]. Moreover, a body condition score (BCS) was determined for each dog based on a 9-point scale [28].

All dogs fasted for 8 to 12 h before blood sampling, and water was offered at libitum. Females were not lactating, not pregnant, and not in oestrus. Non-fasted dogs, dogs on medication at the time of blood sampling or when significant illness was suspected based on history and observation were excluded from the study. Dogs needed to be free of medication for at least 2 months before inclusion. Preventive medication (vaccination, deworming) was allowed until 2 wk. before the consultation [2, 6, 9]. Dogs older than 6 years of age were not included. In addition, the group of adult dogs did not include large-sized dogs older than 5 years of age, that is, dogs in the senior stage, based on previous studies [27]. All dogs selected for this study were privately owned. All owners signed an informed consent form (Additional file 1).

### Blood collection

Samples of 5 ml of blood were collected using venipuncture jugular tubes with vacuum and coagulation activator (BD Vacutainer; BD Medical Technology, Franklin Lakes, NJ). No type of anaesthesia or sedation was used when taking the blood sample. Separation of serum was performed via centrifugation (Ultra-8 digital, LW Scientific, Lawrenceville, GA) from 5 to 10 min at 2500 RPM (846 RCF) when coagulation occurred at room temperature within the first hour after blood collection. The serum was transferred to a 1.5-ml tube (Eppendorf, Hamburg, Germany). Biochemical analysis was performed the same day; when it was not possible to perform analyses on the same day, the serum was frozen at − 20 °C (− 4 °F) and protected from light until analysis the next day, avoiding several cycles of freezing and thawing [29].

### Biochemical analysis of blood samples

We analysed the blood samples obtained in the Laboratory of Diagnostic Pathology using a BTS-350 spectrophotometer (BioSystems, Barcelona, Spain) and reagents (Pointe Scientific, Canton, MI). The analysis was performed according to the manufacturer’s instructions and utilizing standardized methods like shows the Table 2.

**Table 2:** Analytical methods for serum biochemistry using spectrophotometry

Analyte	Method/Principle	Reaction type	Reading mode	Type of reaction	Wavelength
AST	Modified IFCC	Kinetic	–	Decreasing	340 nm
ALT	Modified IFCC	Kinetic	–	Decreasing	340 nm
LDH	Modified IFCC	Kinetic	–	Increasing	340 nm
ALP	p-NPP	Kinetic	–	Increasing	405 nm
GGT	GLUPAC	Kinetic	–	Increasing	405 nm
Total protein	Biuret	Endpoint	Monochromatic	Increasing	540 nm
Albumin	BCG	Endpoint	Monochromatic	Increasing	630 nm
Cholesterol	Enzymatic	Endpoint	Monochromatic	Increasing	500 nm
Triglycerides	Enzymatic	Endpoint	Monochromatic	Increasing	500 nm
Glucose	Enzymatic	Endpoint	Monochromatic	Increasing	500 nm
Urea	Urease, GLDH	Fixed time	–	Decreasing	340 nm
Creatinine	Jaffe, acid picric	Kinetic	–	Increasing	510 nm

*ALP* alkaline phosphatase; *ALT* alanine aminotransferase; *AST* aspartate aminotransferase; *BCG* bromocresol green dye binding; *GGT* gamma-glutamyl transferase; *GLDH* glutamate dehydrogenase; *GLUPAC* Lγ-glutamyl-3-carboxy-4-nitroanilide; *IFCC* International Federation of Clinical Chemistry; *LDH* lactate dehydrogenase; *p-NPP* ρ-nitrofenil phosphate

Monitoring recommendations for clinical chemistry are addressed in the general American Society for Veterinary Clinical Pathology (ASVCP) quality assurance and laboratory standards guidelines [23, 24]. We evaluated the functioning of the analytical instrument with calibration curves for each blood analyte in GraphPad Prism version 6 (GraphPad Software, La Jolla, CA). In addition, the spectrophotometer BTS-350 (BioSystems, Barcelona, Spain) has an internal quality control system based on the Levey-Jennings chart; this analysis allowed us to apply the Westgard rules. Before analysing each determination, the analytical methods were calibrated according to the manufacturer’s instructions with the help of a chemical calibrator and commercial controls (levels I and II) (Pointe Scientific, Canton, MI) [23]. Lipaemic, haemolyzed, and icteric blood samples were excluded from the biochemical analysis [2]. Measurements included in the biochemical analyses were the enzymatic activity of AST, ALT, LDH, GGT, and ALP and the concentrations of cholesterol, triglycerides, total proteins, albumin, globulins, glucose, urea, and creatinine (Table 2). Globulins were determined by subtracting the albumin concentration from the total protein concentration [5, 30].

### Statistical analysis

Statistical analysis was performed with Minitab 17 (Minitab Statistical Software, State College, PA); *p* < 0.05 was considered significant. We evaluated the distribution of the variables by examining the histograms and using a goodness of fit test (Anderson–Darling) [31]. To determine if the variance of two or more groups was significantly different, we used a test of equality of variances with multiple comparisons and Levene’s methods. These methods are valid in non-normal distributions, while in normal distributions, the Bartlett test is used. All tests of variance used a confidence level of 95% [32].

Statistical analyses employed analysis of variance (ANOVA) with a general linear model (GLM), which allows the comparison of multiple factors at two or more levels (*p* < 0.05). Biochemical analytes, body temperature, heart rate, and respiratory rate represent response variables (dependent), while age, sex, and body size represent factors (independent variables). The interaction between factors was also evaluated. When the data did not meet the normality assumptions and homoscedasticity, we performed a Box-Cox transformation using Minitab’s optimal lambda (λ) with a confidence level of 95%. Subsequently, a multiple comparison method (Tukey) was performed with a confidence level of 95% [33].

c using the software reference value advisor (RefValAdvV.2.1, http://www.biostat.envt.fr/reference-value-advisor/) based on the recommendations of the International Federation of Clinical Chemistry (IFCC) and the clinical and laboratory standards institute (CLSI) [22]. This software detected potential outliers by visual inspection of the histograms and dot plots of the reference values and by using the simple Dixon test, which identifies an extremely high or low value as a potential outlier if its distance from the next, more central value in the distribution exceeds one-third of the total range of values [2, 22]. If a blood specimen contributed to more than one outlying observation, all results from that specimen were excluded, as this could possibly indicate subclinical disease [5]. Therefore, the values obtained from the blood specimen of seven dogs were excluded from the statistical analysis because a subclinical disease was detected

## Supplementary Information

**Additional file 1.**

## Data Availability

The datasets used and/or analysed during the current study are available from the corresponding author on reasonable request.

## Abbreviations

ALP: Alkaline phosphatase
ALT: Alanine aminotransferase
ANOVA: Analysis of variance
AST: Aspartate aminotransferase
bpm: beats per minute and breaths per minute
ASVCP: American Society for Veterinary Clinical Pathology
BCG: Bromocresol green dye binding
CI: Confidence interval
G: Gaussian
GFR: Glomerular filtration rate
GGT: Gamma-glutamyl transferase
GLDH: Glutamate dehydrogenase
GLUPAC: Lγ-glutamyl-3-carboxy-4-nitroanilide
IFCC: International Federation of Clinical Chemistry
IQR: interquartile range
LDH: Lactate dehydrogenase
LL: Low limit
NG: Not Gaussian
p-NPP: ρ-nitrofenil phosphate
SD: Standard deviation
UL: Upper limit
