# Supplementary material for: Effect of age, sex, and body size on the blood biochemistry and physiological constants of dogs from 4 wk. to > 52 wk. of age
Source: BMC Vet Res. 2021 Aug 6;17:265. doi: 10.1186/s12917-021-02976-w (PMC8349075; doi:10.1186/s12917-021-02976-w)
Supplement: Supplementary file 1 — Additional file 1. [file 12917_2021_2976_MOESM1_ESM.pdf]

UNIVERSIDAD AUTÓNOMA DE AGUASCALIENTES

Detection of apparently healthy puppies, young and adult dogs:

Questionnaire for owners (Appendix 1).

1) How would you describe your dog's general health? (Mark the correct answer).

☐Very good ☐Good ☐Fair ☐Bad ☐Very bad

2) Living environment (check the correct answer).

☐City ☐Field

3) Lifestyle (check one or more correct answers).

☐ Daily walks ☐ Released regularly in the garden

☐ Stays outdoors ☐ Only indoors

☐ May decide to go outside ☐ Other:

4) Your dog is?

☐ Active ☐ Passive (quiet / little activity)

Has this changed compared to some time ago (weeks, months, or years ago)? ☐No ☐Yes

If your answer is affirmative, specify what changes have you observed?

5) How many walks do you take with your dog and what is the average distance?

6) Have you noticed any change in behavior?

☐No ☐Yes, explain

7) Are there other pets in the home? If so, which one and how many?

☐No ☐Yes, explain

8) Does your dog eat normally? Less or more than before? If it is not normal, please describe.

9) Does your dog drink normally? Less or more than before? If it is not normal, please describe.

10) Is your urine normal? Is the position normal? You. Do you have the impression that it is difficult or painful for your dog to urinate?

11) Is defecation normal? Is the position normal? You. Do you have the impression that it is difficult or painful for your dog to defecate?

12) Has your dog vomited recently? ☐No ☐Yes

When and how often?

Description:

Was any treatment given, which one?

13) Has your dog recently suffered from diarrhea? ☐No ☐Yes

When and how often?

Description:

Was any treatment given, which one?

14) Has your dog had a cough, sneeze or runny nose? ☐No ☐Yes

When and how often?

Description: ☐Productive (phlegm or mucus). ☐ At rest.

☐Not productive (dry). ☐ During exercise.

Was any treatment given, which one?

15) Do you have the impression that your dog is fatigued faster during (moderate) exercise?

☐No ☐Yes, please explain.

16) Does your dog show a fast-breathing pattern and / or shortness of breath? ☐No ☐Yes

If yes, when? ☐During break and / or ☐During activity

17) What food does your dog get? (brand + type) you can select more than one answer.

☐ Commercial foods (croquettes):

☐ Canned food:

☐ Homemade diet:

☐ Raw meat:

What amount?

How many times a day or is it at will (Ad libitum)?

Recent changes in food? ☐No ☐Yes (when?)

Do you receive anything extra (prizes, baits...)? ☐No ☐Yes

18) Is your dog's weight stable? Please specify

☐No ☐Yes

If the answer is no, specify if you have noticed that it increases or decreases ...

19) Has your dog received any medications in the last two months?

☐No ☐Yes

What product, when, how long?

20) Are vaccinations and deworming done regularly? ☐No ☐Yes

When was the last time?

21) Does your dog receive treatment for fleas, ticks, ...? ☐No ☐Yes

When was the last time?

22) Do you brush your dog's teeth? ☐No ☐Yes

If so, how often?

23) Has your dog ever been out of Aguascalientes? ☐No ☐Yes

Where and when?

24) Does your dog have any important medical history? (illness, surgery, trauma, medication)

25) When was his last visit to the vet? How often does a vet see him?

26) Has the dog been previously examined for hypertension or heart murmur?

☐No ☐Yes When?

Which it was the result?

27) Has the dog previously had a blood test?

☐No ☐Yes When?

What test (s)?

Which it was the result?

28) Has an echocardiography (heart ultrasound) been previously performed?

☐No ☐Yes When?

Which it was the result?

Medical history format

(Animal information form)

Number:	Date:

**OWNER INFORMATION**
Owner's name:
Address:
Phone:
Mail:
**PATIENT'S DATA**

Name:	Date of birth and age:	Sex: H/M	Breed:	Weigth:	Size:

Particular signs:

Vaccination: yes / no<br>Date:	Deparasitation: yes / no<br>Date:	Is your dog spayed? yes / no
Type of food	Fast: yes / no<br>8h 12h	Origin<br>rural / urban

Anamnesis:

T°	Heart rate	Pulse	Mucous	Capillary fill time<br>*seg*
Breathing frequency	Palmopercusión	Tusigen reflex	Swallowing reflex	Body condition
Hydration	Lymphonodes	Abdominal palpation	Attitude	

Observations (eyes, ears, skin and annexes, locomotion, skeletal muscle, nervous, cardiovascular, respiratory, digestive, genitourinary system):

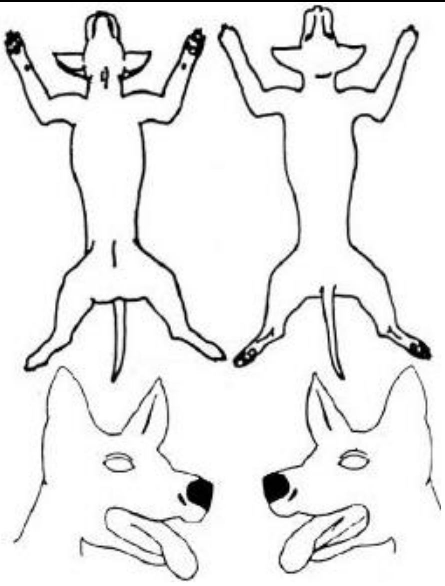

Previous illnesses or procedures:

Informed consent

I am the owner or representative of the pet owner and I have the authority to execute this consent, therefore, I give my authorization for the named pet to undergo a clinical examination and take a blood sample (jugular or cephalic vein) to perform laboratory tests (blood chemistry) for free as part of the research project of the MVZ Ana Luisa Montoya Navarrete thesis of the Autonomous University of Aguascalientes. I understand that necessary precautions will be taken to reduce stress and safeguard my pet's health during the procedure.

My signature below indicates that I have read and understand this consent.

Signature: Date:
